# Initial Studies on the Effect of the Rice–Duck–Crayfish Ecological Co-Culture System on Physical, Chemical, and Microbiological Properties of Soils: A Field Case Study in Chaohu Lake Basin, Southeast China

**DOI:** 10.3390/ijerph20032006

**Published:** 2023-01-21

**Authors:** Jun Yan, Jingwei Yu, Wei Huang, Xiaoxue Pan, Yucheng Li, Shunyao Li, Yalu Tao, Kang Zhang, Xuesheng Zhang

**Affiliations:** 1 School of Resources and Environmental Engineering, Anhui University, Hefei 230601, China; 2 Anhui Province Key Laboratory of Wetland Ecosystem Protection and Restoration, Anhui University, Hefei 230601, China

**Keywords:** rice–duck–crayfish co-culture, soil quality, nitrogen and phosphorus losses, heavy metals, eco-environmental profits

## Abstract

Rice–duck and rice–crayfish co-culture patterns can increase soil productivity and sustainability and reduce the use of chemical pesticides and fertilizers, thereby reducing the resulting negative environmental impacts. However, most studies have focused on the rice–duck and rice–crayfish binary patterns and have ignored integrated systems (three or more), which may have unexpected synergistic effects. To test these effects, a paddy field experiment was carried out in the Chaohu Lake Basin, Hefei city, Southeast China. Four groups, including a rice–duck–crayfish ecological co-culture system (RDC), idle field (CK), single-season rice planting system (SSR), and double-season rice planting system (DSR), were established in this study. The results showed that the RDC improved the soil physical properties, fertility, humus content, and enzyme activity. In the RDC system, the soil total nitrogen content ranged from 8.54% to 28.37% higher than other systems in the 0-10 cm soil layer. Similar increases were found for soil total phosphorus (8.22–30.53%), available nitrogen (6.93–22.72%), organic matter (18.24–41.54%), urease activity (16.67–71.51%), and acid phosphatase activity (23.41–66.20%). Relative to the SSR treatment, the RDC treatment reduced the total losses of nitrogen and phosphorus runoff by 24.30% and 10.29%, respectively. The RDC also did not cause any harm to the soil in terms of heavy metal pollution. Furthermore, the RDC improved the yield and quality of rice, farmer incomes, and eco-environmental profits. In general, the RDC can serve as a valuable method for the management of agricultural nonpoint-source pollution in the Chaohu Lake area and the revitalization of the countryside.

## 1. Introduction

As a major food crop, rice is planted worldwide, especially in Asia, and its annual planting area is more than 160 million hectares [1]. With the rapid growth of the population over the past few decades, chemical fertilizers and pesticides have been extensively used to increase the productivity of agriculture and meet the demand for production [2]. However, excessive use of chemical fertilizers causes negative impacts on the environment, such as damage to the soil structure, reduction in soil fertility, emissions of greenhouse gases, and pollution derived from agricultural nonpoint sources [3,4,5]. Meanwhile, only 20–50% of the nitrogen and 30%-45% of the phosphorus in fertilizers can be utilized by crops, and the rest is converted into environmental waste [6]. In such cases, rice–livestock co-culture techniques (e.g., rice–fish [7], rice–crayfish [8], rice–frog [9], rice–crab [10], and rice–duck [11]) have received wide attention. Co-culture of rice and animals has been suggested as a method to improve the utilization of water and land resources to provide both rice and meat for humans, while reducing the risk of environmental pollution related to rice production [12,13].

The rice–crayfish co-culture technique is a type of rice–aquatic animal co-culture system with environmental advantages [8]. Paddy fields are an appropriate habitat for aquatic animals, and the crops provide shelter for aquatic animals from sunlight and bird attacks [14]. Additionally, aquatic animals prey on pests in paddy fields, which reduces the feed cost of crayfish and pesticide usage [15]. Moreover, the excrement of crayfish provides organic fertilizer for rice, and conversely, the high water quality requirements of crayfish further limit the use of pesticides and fertilizers [16]. Hu et al. [17] pointed out that this co-culture system improves not only soil fertility and nutrient utilization but also enzyme activity. Xu et al. [18] suggested the co-culture system will increase cost but will substantially improve economic efficiency.

The rice–duck co-culture model is a traditional ecological system in China that dates back to the Ming Dynasty [19,20]. During the process of co-culture, ducks peck at weeds and pests among the rice plants in the daytime until the rice tassels. In addition to the aforementioned merits of the rice–crayfish system, this system also has a positive effect on controlling rice diseases, weeds, and pests [21]. Furthermore, the emission of CH_4_ in the soil is concurrently reduced [19]. Compared to traditional rice farming methods, these co-culture approaches can (1) improve the utilization capacity of soil and reduce nonpoint-source pollution, (2) produce both rice and meat for human consumption and gain great economic benefits for farmers, and (3) reduce the health risks triggered by chemical fertilizers and pesticides [5,22,23]. However, most previous studies have focused on the two-component co-culture pattern and less on systems integrating more than two-components, which may have potential synergistic effects. The effects of such co-culture models on soil humus, nitrogen and phosphorus runoff, and heavy metal (HM) concentrations in rice fields have not yet been thoroughly explored. In addition, the environmental impact of the rice-duck-crayfish co-culture system has not been studied.

Chaohu Lake is one of the five largest freshwater lakes in China, and eutrophication caused by large amounts of nitrogen and phosphorous has attracted widespread concern [24]. There is 800 hm^2^ of farmland around Chaohu Lake, and the rice fields are mainly planted in a traditional monoculture model (single-season or double-season rice cultivation) [25]. Consequently, large amounts of chemical pesticides and fertilizers are utilized to obtain high rice yields and are major nonpoint-source pollution contributors to eutrophication and organic pollution in Chaohu Lake. In Chaohu Lake, the primary pollutants that exceeded the standard [26] were nitrogen and phosphorous, of which 60–70% were derived from agricultural nonpoint-source pollution [27,28]. Therefore, the development of suitably integrated rice planting techniques in this area is urgently needed for eutrophication control and economic benefits.

The primary goal of the present study is to evaluate the environmental benefits of the rice–duck–crayfish ecological co-culture system (RDC). The specific objectives of this work are (1) to assess the impacts of the RDC on soil quality via the measurements of basic soil physicochemical properties, humus content, and enzyme activities in field experiments; (2) to determine the system’s ability to manage typical pollutants (e.g., nitrogen and phosphorus runoff losses and soil HM concentrations) through a field study; and (3) to analyze the improvement in rice quality with the RDC pattern, as well as the eco-environmental profits in different co-culture systems. Our study can provide new guidelines for the development of ecological agriculture.

## 2. Materials and Methods

### 2.1. Location of the Study Area

**Figure 1:**
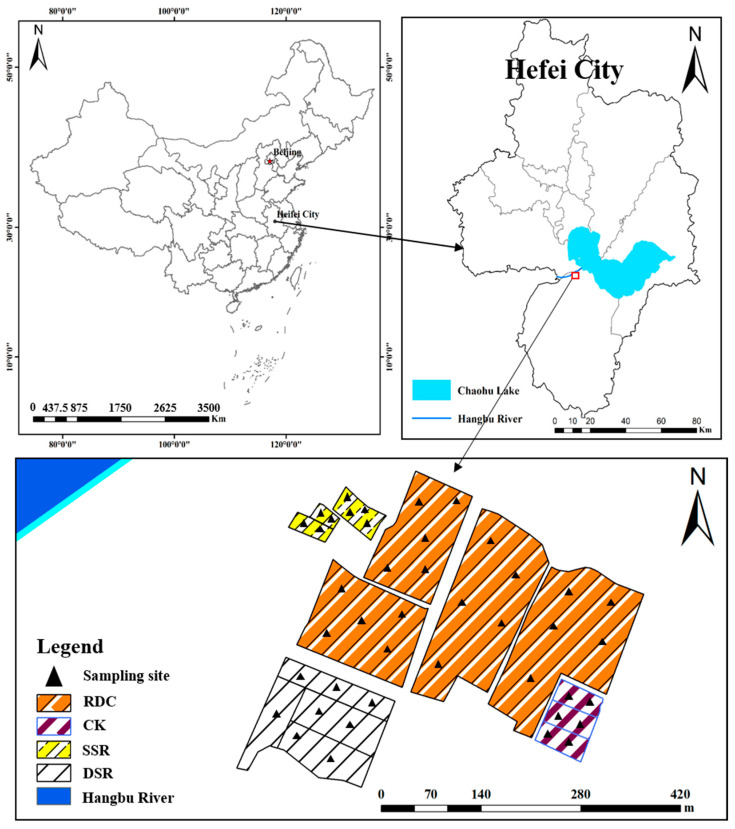
The study area and sampling sites.

The study site was in Beiwei village, Lujiang County, Hefei city, Anhui Province, China (117.14.22–117.14.27° E 31.29.22–31.29.26° N) (Figure 1). The average temperature is 15.8 °C, the average precipitation is 1188.1 mm, and the average frost-free period is 238 days. The field trial was conducted in 2017, and the soil samples were collected in November 2017. The soil sampling sites are illustrated in Figure 1. All experimental fields were left fallow for one year before the experiment. The soil in the field trial was sandy loam. The properties of the surface soil in the paddy field before the trial were as follows: 1.31 ± 0.21 g cm^−3^ bulk density, pH 5.43 ± 0.38, 1440 ± 86 mg kg^−1^ total nitrogen (TN), 1325 ± 105 mg kg^−1^ total phosphorus (TP), 138.80 ± 16.10 mg kg^−1^ alkali-hydrolysable nitrogen (AN), 21.90 ± 2.01 mg kg^−1^ available phosphorus (AP), 107.52 ± 8.15 mg kg^−1^ available potassium (AK), 25.52 ± 2.11 g kg^−1^ soil organic matter (SOM), 11.26 ± 0.45 mg kg^−1^ Cu, 102.20 ± 5.22 mg kg^−1^ Zn, 61.28 ± 1.25 mg kg^−1^ Pb, 16.35 ± 1.40 mg kg^−1^ As, 18.53 ± 0.85 mg kg^−1^ Cr, 0.17 ± 0.01 mg kg^−1^ Cd, 0.88 ± 0.11 mg g^−1^ urease, 0.75 ± 0.06 mg g^−1^ acid phosphatase, and 0.85 ± 0.07 mL g^−1^ catalase.

### 2.2. Materials

The test rice was *Nan Gen 46*, with a total growing period of approximately 158 days. The ducklings used in the present study were Chaohu ducks (*Anas platyhynchos domestica*), which grow fast and have a compact body shape, black and grey feathers, lustrous orange toe webs, and black claws. Crayfish (*Procambarus clarkii*), provided by Anhui Yi Gaofeng Co., Ltd. (Hefei, China), are omnivorous and have a high growth rate, adaptability, and feeding range, including aquatic plants. The compound fertilizer (N:P_2_O_5_:K_2_O = 21:9:10) was supplied by CNSG Anhui Hong Sifang Co., Ltd. (Hefei, China), and urea with a nitrogen content of 46% was produced by Anhui Haoyuan Chemical Group Co., Ltd. (Fuyang, China).

### 2.3. Design of Field Experiments

**Figure 2:**
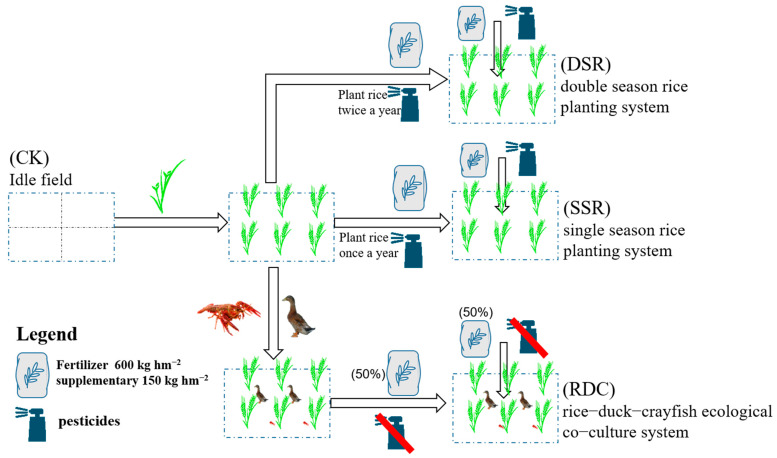
The general scheme of the field experimental designs.

Four treatment groups were established based on the application of basal fertilizer involving a compound fertilizer (N:P_2_O_5_:K_2_O = 21:9:10, 600 kg hm^−2^) and supplementary fertilizer (urea, 150 kg hm^−2^). These treatments were the idle field (CK), single-season rice planting (SSR), double-season rice planting (DSR) and rice–duck–crayfish ecological co-culture system (RDC) (Figure 1 and Figure 2). In the Chaohu Lake Basin, excessive use of chemical fertilizers is one of the important causes of agricultural nonpoint-source pollution. Therefore, this experiment will reduce the chemical fertilizer in the RDC system by 50%. The topography of the study area was limited. Although the areas were different, each treatment was set up in at least three parallel groups to ensure the accuracy of the experiment. Moreover, we made the area of the RDC system as large as possible, to evaluate whether it is suitable for large-scale rollout in the future. At least three replicates of each planting pattern were conducted, and the specific experimental setup is shown in Table 1.

### 2.4. Field Management

#### 2.4.1. RDC Group

**(1) Preparation stage**: A circular ditch (2.5 m wide and 2 m deep) was dug around the field. Quicklime and sodium humate were used in the field. Fourteen days later, the drug toxicity disappeared, and *Hydrilla verticillata* and native weeds were planted in the ditches for crayfish consumption. An anti-fleeing net and a ridge were set up around the field, and a duck shed was set up. Some feed and grains were offered in the duck house to feed the ducks.

**(2) Co-culture stage**: On 1 July 2017, the field was tilled and properly stocked with crayfish fry (375 kg hm^−2^). Rice seedlings were transplanted into the field on 3 July with a (plant × row) spacing of 20 × 20 cm, and basal fertilizer (300 kg hm^−2^) was applied on 3 July. On 11 July, supplementary fertilizer (75 kg hm^−2^) was applied. On 13 July, ducklings (195 ducks hm^−2^) were placed in the field. The water levels in the field were maintained at 8–10 cm during the rice–duck cycle. Ducks were driven out of the field during the rice tasseling period on 11 September, and the rice was harvested on 14 November 2017. While the rice was being harvested, crayfish were caught in the field.

#### 2.4.2. Other Groups

**CK**: No rice, ducks, crayfish, fertilizers, or pesticides were included in this group. The water levels in the field were maintained at 8–10 cm.

**SSR and DSR**: No ducks or crayfish were placed in these two field trials. According to the local planting pattern, the basal fertilizer (600 kg hm^−2^) and supplementary fertilizer (150 kg hm^−2^) were used for one season. Pesticides were sprayed four times and herbicides twice during the growth period of the rice. The other procedures were exactly the same as those used for the RDC.

### 2.5. Sample Collection

After the rice harvest, soil samples (0–20 cm) were collected using the *S*-shaped and 5-point collection method. Plant root remnants and small rocks were removed, and the samples were well mixed and divided into two equal portions. One portion of fresh soil was passed through a 2 mm sieve (10-mesh) and stored at 2–4 °C for further use in the measurement of enzyme activity. Meanwhile, the other portion of the soil was air-dried and either sieved through a 100-mesh screen for nutrient quantification or used for analysis of the humus content.

Field runoff collection was performed in the RDC and SSR treatments (because only these two groups have the same rice-growing period), and standard runoff cells were used during rainfall. The runoff cells were 5 m × 10 m (width × length). Bricks were used to build the boundaries, and a waterproof film was laid on the top of each runoff cell. After recording the height of the water in the cell, the runoff pond drainage valve was opened to drain the water for the next collection. Surface water samples were collected from each treatment area accordingly, and sulfuric acid (ρ = 1.84 g mL^−1^) was added to the water bottles to adjust the pH to <2.0. Samples were brought back to the laboratory within 4 h, and then water quality indicators (TN, TP, dissolved total phosphorus (DTP), NH_4_^+^-N, and NO_3_^−^-N) were analyzed immediately.

Rice samples of the RDC and SSR treatments were collected. Specifically, 40 plants from each field were randomly selected at the maturity period, and their heights were recorded. Among them, ten plants with heights close to the average plant height were harvested and transported to the laboratory for analysis of rice quality.

### 2.6. Chemical Analysis

Soil bulk density and total porosity were determined using the ring knife method [29]. The samples were air-dried, and the soil chemical properties (pH, SOM, TN, TP, AN, AP, and AK) were measured according to reported methods[30]. The detailed methodology can be found in the Supplementary Materials (SM). The soil humus fractions were extracted and separated using the humus composition modification method [31], and were measured in a TOC analyzer (liquiTOCII, Elementar, Germany). Determination of the optical properties of humic substances was performed using a UV spectrophotometer (UV-360, Shimadzu, Japan). Enzymatic activity was measured according to the descriptions reported by Guan et al. [32], and the detailed procedures are described in the SM. The HM contents were determined using an inductively coupled plasma mass spectrometer (Agilent 7500 Series, Agilent, CA, USA), and the detailed procedures are shown in the SM. A three-step sequential extraction procedure from modified BCR [33] was applied to extract the four fractions of HMs investigated, and the detailed extraction procedures can be found in the SM.

The TP and DTP levels in the water samples were determined using the ammonium molybdate spectrophotometric method[34]. The TN concentration was determined using the alkaline potassium persulfate digestion method[35], and the detailed methodology can be found in the SM. The NH_4_^+^-N concentration was determined using Nessler’s reagent spectrophotometry[36], and the NO_3_^−^-N concentration was determined using UV spectrophotometry (UV-360, Shimadzu, Japan).

For rice quality, the brown rice rate, milled rice rate, head milled rice rate, chalky kernel rate, chalkiness, length–width ratio, gel consistency, alkali spreading value, amylose content, and protein content of the rice were measured according to the methods described in NY/T 83-2017 Determination of rice quality [37].

### 2.7. QA/QC

The chemicals used in the experiments were all of analytical grade. All glassware used for analysis was soaked overnight in 10% HNO_3_ and rinsed thoroughly with deionized water before use. To ensure the accuracy of the data, three parallel samples were analyzed during the determination process for quality control. The accuracy of the data was checked using materials conforming to national standards (GBW-07404), and the recoveries of each HM in the duplicates and reference materials ranged from 95.00% to 115.00%. The relative standard deviation of duplicates was better than 5% for the total content determination of each HM and was less than 10% for the BCR-extracted results.

### 2.8. Data Processing and Statistical Analysis

All experimental significant differences were determined using SPSS 26.0 (SPSS Inc., Chicago, IL, USA). Prior to performing statistical analysis on the data, Shapiro–Wilk tests were applied to examine the data normality. One-way analysis of variance (ANOVA) with Duncan’s multiple comparisons test was used to determine significant differences (*p* < 0.05) between different groups. All data are shown as the mean ± sd. All figures in this study were prepared using Origin 2021 software (Origin Lab Inc., Northampton, MA, USA).

## 3. Results and Discussion

### 3.1. Improvements of the RDC Treatment on the Soil Quality

#### 3.1.1. Physicochemical Properties

The field experiment results indicated improvements in the physical properties after co-culturing (Table S4). With increasing soil depth, the soil bulk density gradually increased, and the total porosity gradually decreased in all planting patterns (*p* < 0.05) (Table S4). Such results suggested that the RDC treatment could reduce soil bulk density and increase soil porosity. This might be attributed to crayfish having a strong burrowing ability, and the burrows they dig can provide some space [38]. The burrowing activity of benthic animals not only reduces soil bulk density and increases soil porosity, but also increases the water infiltration rate [39]. In addition, the uninterrupted foraging activities of ducks also have a positive effect on loosening the soil [40].

**Figure 3:**
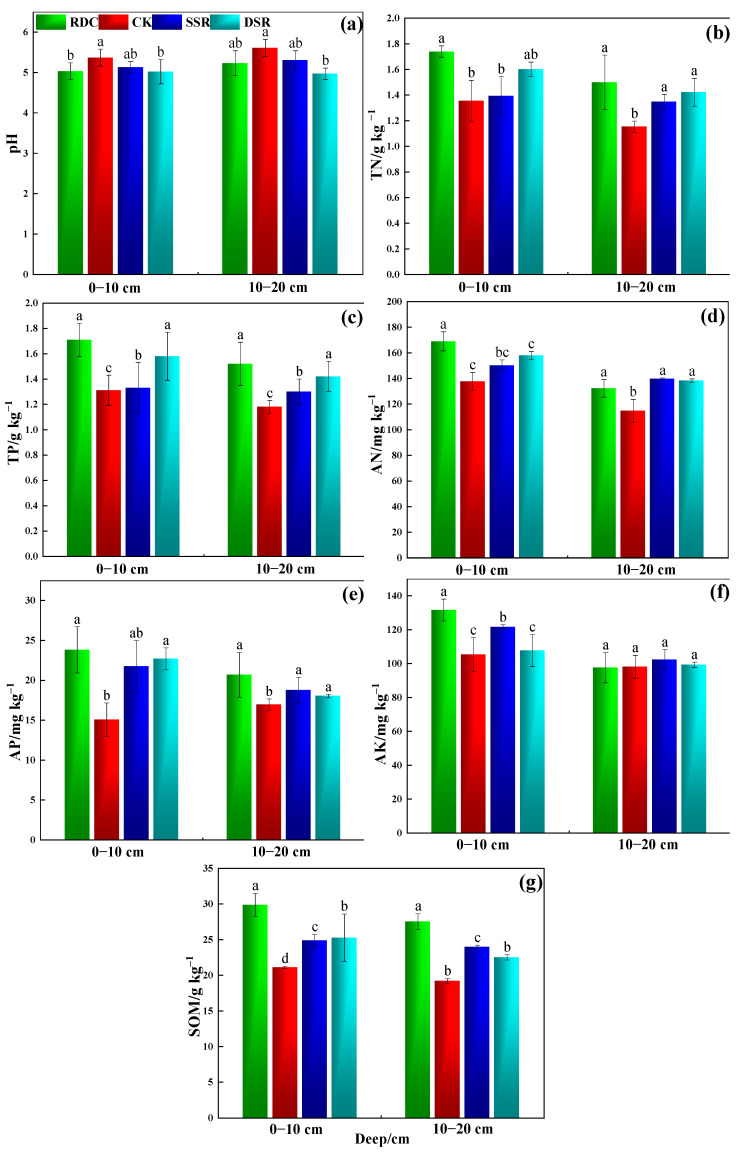
Effects of different planting patterns on the chemical properties of the soil. RDC: rice–duck–crayfish ecological co-culture system, CK: idle field, SSR: single-season rice planting system, DSR: double-season rice planting system. (**a**) pH, (**b**) TN is total nitrogen, (**c**) TP is total phosphorus, (**d**) AN is alkali-hydrolysable nitrogen, (**e**) AP is available phosphorus, (**f**) AK is available potassium, and (**g**) SOM is soil organic matter. Small letters show the significant difference.

The field study results showed significant improvements in nutrient status with the RDC treatment. The soil pH showed an increasing trend with the deepening of the tillage layer, and there were significant differences between the different cropping patterns (*p* < 0.05) (Figure 3a). In the 0–20 cm soil layer, the maximum TN content was found in the RDC group, while the minimum TN content was recorded in the CK group (Figure 3b). For the 10-20 cm soil layer, the TN was slightly higher in the RDC group (*p* > 0.05) than in the SSR and DSR groups. Similar trends in the TP contents with the different treatments were also recorded (Figure 3c).

In the 0–10 cm soil, the highest AN, AP, and AK contents were found in the RDC, and the lowest AN, AP, and AK contents were found in the CK (Figure 3d–f). The soil AN, AP, and AK in the 10–20 cm soil layer of the RDC were not significantly different from those of the other systems. Compared to the CK and DSR, the RDC significantly enhanced the SOM content, with relative increases of 8.77 and 3.61 g kg^−1^ in the 0–10 cm soil layer, respectively, and 6.04 and 4.01 g kg^−1^ in the 10–20 cm soil layer, respectively (Figure 3g). These results showed that the RDC treatment could improve soil chemical properties, e.g., TN, TP, AP, AN, AK, and SOM, after one season of rice cultivation, which is consistent with the results of previous studies [11,21]. However, unlike the above studies, this study found that RDC treatment was better for the improvement in surface soil chemical properties (1–10 cm).

A possible reason for the higher soil nutrient content in the RDC was that duck activities in the field (e.g., pecking, trampling, and shaking rice plants) could promote oxygen exchange between the soil and the water, thus increasing the nutrient uptake of the rice [41]. Ducks move freely and excrete feces randomly in the field, leading to direct fertilization on the field surface [11]. Another reason is that crayfish feeding and excretion (rich in organic matter) can enhance the content of nutrients in the soil, which can elevate the population of microorganisms and accelerate the circulation and activation of soil nitrogen. In addition, crayfish feeding activities promote the efficient use of soil nitrogen by disturbing the release of fixed nitrogen nutrients in the soil and improving the redox state of the soil, which, in turn, promotes N mineralization as well as nitrification [17]. Moreover, our findings were consistent with those of Li et al. [21], who found higher soil fertility at maturity in rice–duck co-culture systems than conventional rice cropping systems. Additionally, Yuan et al. [42] also reported that the rice–crayfish co-culture system could significantly improve the soil quality in paddy fields.

#### 3.1.2. Soil Humic Substances

**Figure 4:**
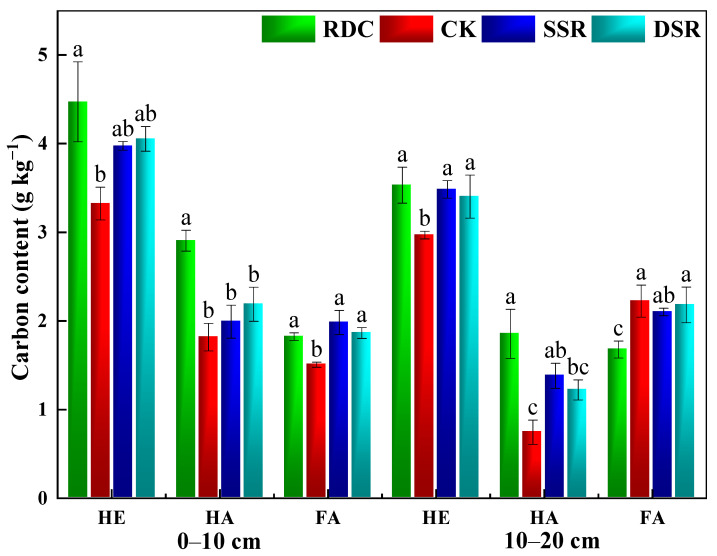
Effects of different planting patterns on the soil humus level and its components. HE, extractable humus; HA, humic acid; FA, fulvic acid. Small letters show the significant difference.

Humus substances play a crucial role in maintaining soil fertility, such as improving soil quality, and maintaining the balance of soil carbon pools [43]. As shown in Figure 4, the extractable humus (HE) contents in the 0–10 cm soil layers of the RDC (34.64%), SSR (19.58%), and DSR (21.99%) were significantly higher than those of the CK. Similar trends of increased HE were also found in the 10–20 cm soil layer.

The E4/E6 ratio and Δlogk values are the optical properties that indicate the molecular complexity of soil humic acid (HA) [44]. The HA-E4/E6 values were ranked in the order of SSR < DSR < CK < RDC. Among them, the HA-Δlogk values of the four treatments also showed significant differences (*p* < 0.05) (Table S5). The results showed that the RDC promoted the condensation, aromaticity, and molecular simplification of HA in soils, thereby reducing the structural complexity of HA.

**Figure 5:**
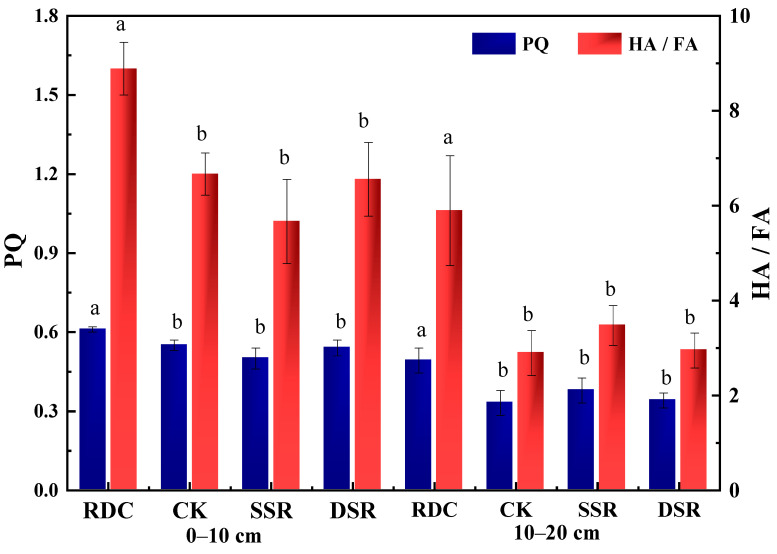
Improvements of different patterns on the structural characteristics of soil humus. HA, humic acid; FA, fulvic acid; HE, extractable humus; PQ, HA/HE. Small letters show the significant difference.

HA/FA indicates the degree of humus condensation in soil; the higher the value is, the more active and complex the soil humus structure [45,46]. The PQ value (HA/HE) represents the relative formation rate and the conversion relationship between humic acid and humus in the soil [47]. The trends of the PQ value and HA/FA value were found to be RDC > CK > DSR > SSR in the 0–10 cm layer. In the 10–20 cm layer, the PQ values (RDC > SSR > DSR > CK) and HA/FA values (DSR > RDC > SSR > CK) showed different changes (Figure 5). The RDC enhanced the humification process and stability of the soil and also promoted the relative formation rate and the conversion relationship between humic acid and humus, thus improving the activity of humus in the soil [48].

#### 3.1.3. Soil Enzyme Activity

**Figure 6:**
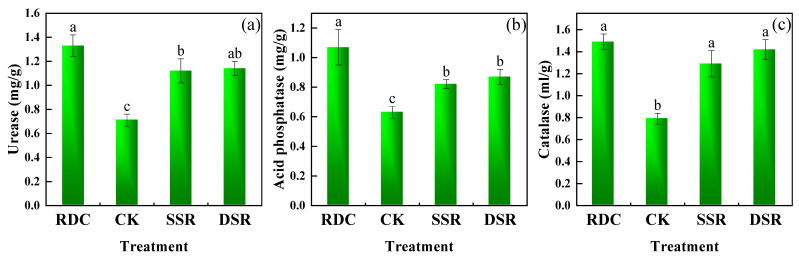
Effects of different planting patterns on soil enzyme activity. (**a**) Urease, (**b**) Acid phosphatase, (**c**) Catalase. Small letters show the significant difference.

The soil urease, acid phosphatase, and catalase activities of different treatments were measured (Figure 6). Compared to those of the CK, SSR, and DSR, the urease and acid phosphatase catalase activities of the RDC were significantly higher (*p* < 0.05), by 87.51%, 18.39%, and 16.67% and by 70.21%, 30.76%, and 23.41%, respectively. For soil catalase activity, although significant differences (*p* < 0.05) were observed between the RDC, SSR, and DSR and the CK, no significant differences were observed between the three treated groups. This result suggests that the integrated RDC treatment was able to elevate the soil enzyme activities efficiently. This might be because the duck and crayfish manures served as organic fertilizers in the RDC, thus increasing soil enzyme activities[49,50].

### 3.2. Effect of RDC and SSR Systems on Nitrogen and Phosphorus Loss via Runoff

During the entire rice-growing period, four major runoff events were recorded in the rice paddies (9 July, 4 August, 14 August, and 6 October), all of which were generated by continuous rainfall (Figure S1).

**Figure 7:**
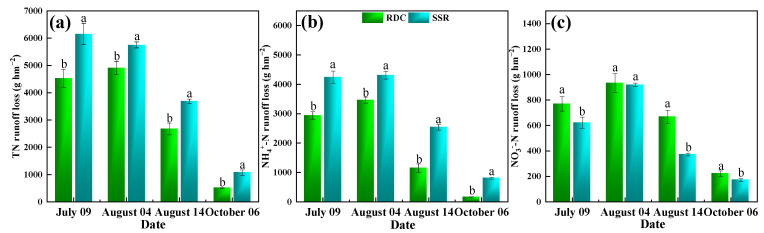
Nitrogen losses via runoff. (**a**) TN is total nitrogen, (**b**) NH_4_^+^-N is ammonium nitrogen, (**c**) NO_3_^−^-N is nitrate nitrogen. Small letters show the significant difference.

The total nitrogen runoff loss from the four runoff events accounted for 35.87–36.95%, 34.50–38.94%, 21.18–22.12%, and 3.99–6.41% of the TN runoff (Figure 7). The TN loss was mainly concentrated in the first two runoff events, and the NH_4_^+^-N loss was higher than the NO_3_^−^-N loss.

**Figure 8:**
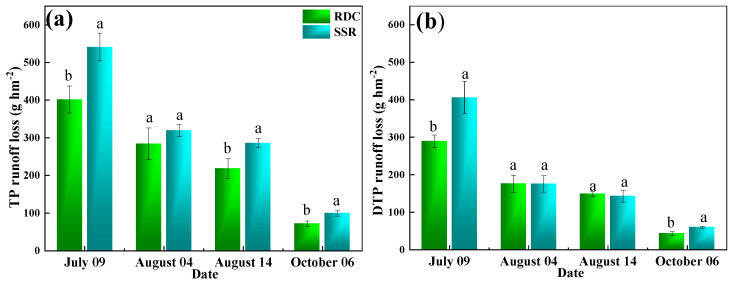
Phosphorus losses via runoff. (**a**) TP is total phosphorus, (**b**) DTP is dissolved total phosphorus. Small letters show the significant difference.

Compared to that in the SSR, the TP loss was lower in the RDC in each runoff event (Figure 8). Specifically, the largest TP loss was present on July 09, which was 0.40 kg hm^−2^ and 0.54 kg hm^−2^ in the RDC and SSR, respectively. The DTP loss of the RDC was higher than that of the SSR in the first and fourth runoff events (*p* < 0.05). The amount of nitrogen and phosphorus runoff loss from paddy fields is closely related to the amount of fertilizer applied, the time of fertilizer application, and the amount of runoff [51]. In this work, the nitrogen and phosphorus runoff from rice mainly occurred in the early stage of fertilization. The closer the runoff event is to the fertilization period, the greater the nitrogen loss in runoff. This might be because after nitrogen application, the nitrogen content of the surface water in the field quickly reaches the maximum value, and at this time, the water level is high, which easily leads to runoff generation, resulting in great losses of nitrogen and phosphorus [52]. With rice growth, large amounts of nitrogen and phosphorus in water are consumed, resulting in significant reductions in the nitrogen and phosphorus in the water. The height of the surface water in the fields in the later stage is lower, making runoff generation more difficult. As a consequence, the nitrogen and phosphorus losses via runoff in the later stage are small [53,54].

Compared to those in the SSR, the TN and TP losses in the RDC were lower by 24.30% and 10.29%, respectively (*p* < 0.05) (Table S6). The NH_4_^+^-N loss and NO_3_^−^-N loss were significantly (*p* < 0.05) reduced by 35.24% and 19.56%, respectively. This indicated that the RDC could control the TN and TP losses from paddy field runoff. Duck and crayfish manure have a slow rate of organic fertilizer nutrient release, which can provide nutrients during the whole growth period of rice, avoiding the concentrated loss of nitrogen nutrients in the early stage and reducing the loss of nitrogen to a certain extent [55]. The nitrogen runoff loss was mainly due to soluble nitrogen (NH_4_^+^-N and NO_3_^−^-N), and the proportion of particulate-state nitrogen was low [56]. The predominant forms of phosphorus in paddy water and soil are mainly the dissolved and mineral forms. Phosphorus loss is mainly influenced by rainfall, soil compactness, and fertilizer application [52]. The proportion of DTP in the RDC was higher than that in the SSR in each runoff period. This is because DTP is the main component of TP in animal manure and orthophosphate, and it can be absorbed by rice directly. The present study demonstrated that the RDC can reduce the loss of nitrogen and phosphorus via runoff from paddy fields, thus providing countermeasures to alleviate and mitigate agricultural nonpoint-source pollution.

### 3.3. Soil HMs in Different Groups

**Figure 9:**
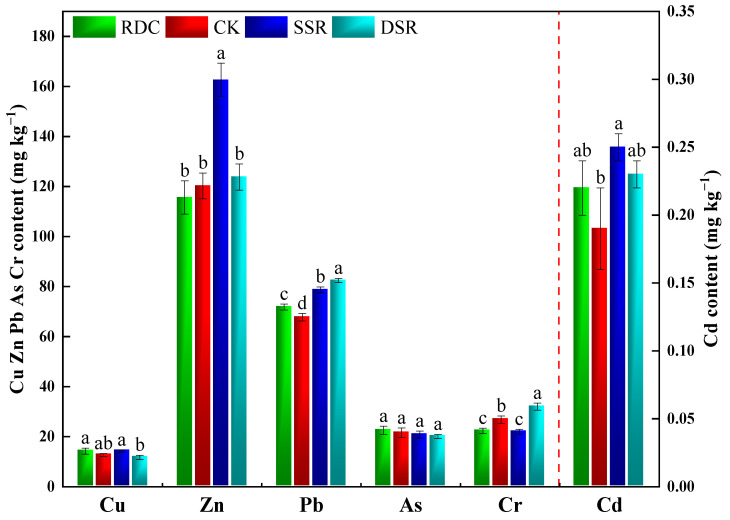
Contents of HMs in the surface soils of paddy fields. Small letters show the significant difference.

The pH values of the surface soils in this study were in the range of 4.96 to 5.61 (mean value: 5.23), indicating a weakly acidic environment in the paddy soils. According to the Soil Environment Quality Risk Control Standard for Soil Contamination of Agriculture Land of China [57], the risk screening values for the soil contamination of agricultural land for Cu, Zn, Cd, Pb, As, and Cr are 500, 200, 0.3, 80, 30, and 250 mg kg^−1^, respectively. The concentrations of typical HMs in the soils of the different culture patterns are presented in Figure 9. The soil HM contents in the four treatments were lower than the intervention values of the soil pollution risk in agricultural land. In general, compared to the conventional cultivation (SSR and DSR) systems, the RDC exhibited lower contents of Zn (28.92%, 6.62%), Pb (8.81%, 12.97%), and Cr (−1.56%, 30.23%) in the soil. This might be due to the presence of HM elements in chemical fertilizers that can accumulate in the soil following fertilization, such as Pb, Ni, and Cr [58]. Therefore, the reduction in chemical fertilizer application led to decreases in the contents of several HMs in the soil. In addition, the RDC exhibited elevated soil Cu content (0.42% and 20.95%), and no significant effects on the soil As and Cd contents were observed. This is mainly because animal feed contains significant concentrations of Cu and Cd, and a large portion of these HMs are excreted through urine and feces [59].

Table 2 shows that the Nemerow index (*P_N_*) of the different cropping patterns were within safe levels, and there were no significant differences between the four patterns. The calculated *P_N_* was lower than 1.0 and exhibited the order of DSR > SSR > RDC > CK. These results indicate that the RDC did not cause any environmental harm via increases in soil HM concentrations.

**Figure 10:**
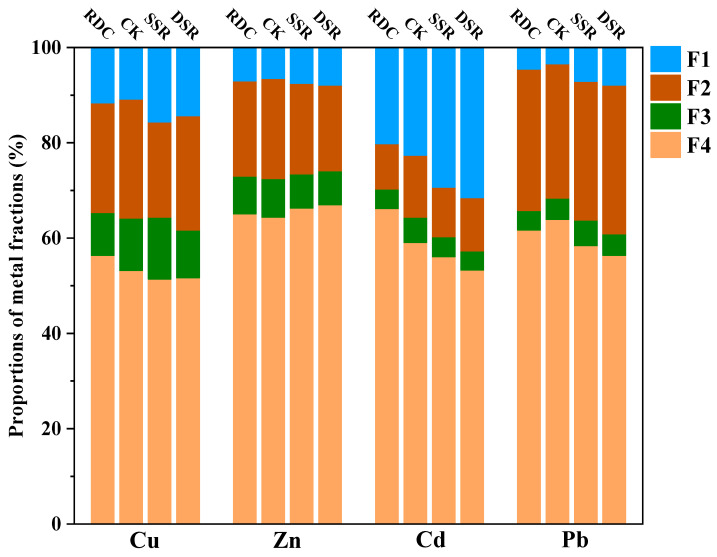
Chemical fractions of HMs in surface soils of paddy fields. F1, the acid-soluble fraction; F2, the reducible fraction; F3, the oxidizable fraction; F4, the residual fraction.

The distributions of Cu, Zn, Cd, and Pb in the four fractions are shown in Figure 10. Compared with those in the conventional rice crop systems (SSR and DSR), the Cu, Cd, and Pb in the RDC had a higher F1 proportion. The F1 proportion of Zn was approximately the same for each system. These results suggest that the RDC can reduce the bioavailability of Cu, Pb, and Cd. On the one hand, the increase in soil pH may be an important reason for the decrease in bioavailability [60]. As the soil pH changes from low to high, the negative charge on the surface of soil colloids increases, leading to greater formation of Fe and Mn oxides in the soil, thus increasing the HM adsorption capacity[61]. On the other hand, the bioavailability of HMs in soil is strongly affected by the SOM content, which can immobilize HMs through adsorption or the formation of a stable organic fraction[62,63]. Humic substances contain a large number of functional groups (e.g., carboxyl and phenolic−OH groups) that play a crucial role in forming complexes with metal ions[64].

### 3.4. Rice Quality Promotion in the RDC System

The brown rice rate, milled rice rate, and head milled rice rate were slightly higher in the RDC than in the SSR (Table S7a). The chalky rice rate and chalkiness degree of the rice produced from the RDC were significantly lower (*p* < 0.05) than those produced from the SSR, but there were no significant differences in grain length, grain width, or length–width ratio between the two systems (Table S7b). The length–width ratio, mainly controlled by genetic factors, is an important index used to evaluate the indica-japonica variety of rice. Unlike the length–width ratio, the milled rice rate, head milled rice rate, and chalkiness are related to environmental factors, and the chalkiness of rice is extremely sensitive to changes in the external environment [65]. Previous studies proved that the frequent activities of ducks in paddy fields can effectively stimulate the root growth of rice plants and are beneficial to rice growth, e.g., by increasing the effective tillering of rice plants, improving the photosynthesis of rice leaves, and promoting better absorption of nutrients by rice from the surrounding environment[66].

Gel consistency and amylose content are closely related to the cooking quality of rice, and rice with a high gel consistency and low amylose content has a better taste [67]. The results suggest that the protein content and gel consistency of the rice from the RDC were significantly higher than those from the SSR (*p* < 0.05), by 8.15% and 6.52%, respectively. In contrast, the amylose content (*p* < 0.05) and alkali spreading value (*p* > 0.05) in rice from the RDC were lower than those from the SSR to different degrees (Table S7c). It is speculated that duck and crayfish manure, acting as additional organic fertilizers, promote the quality of the rice in the RDC. In addition, the RDC treatment also enhances the uptake rates of nitrogen in rice, and nitrogen levels are positively related to the gel consistency of rice, thus improving the taste of the rice. According to the standard for high-quality rice[68], the head milled rice rate and chalkiness degree of the RDC rice meet the standards of Grade 1 rice, and the amylose content is also up to the standard for high-quality rice.

**Figure 11:**
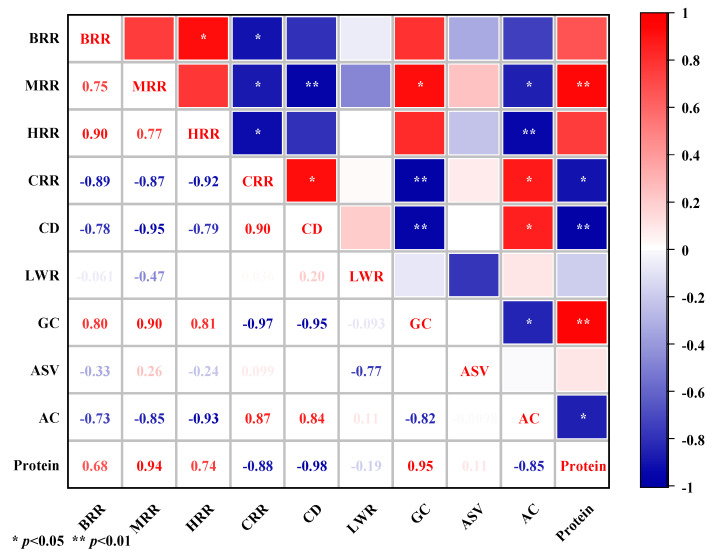
Correlation of rice quality indicators. BRR, brown rice rate; MRR, milled rice rate; HRR, head milled rice rate; CRR, chalky rice rate; CD, chalkiness degree; LWR, length–width ratio; GC, gel consistency; ASV, alkali spreading value; AC, amylose content. The correlations of rice quality indicators in this study were evaluated by using Pearson correlation analysis, with a significance level at *p* < 0.05 or *p* < 0.01. *n* = 40.

The correlations between the rice quality indicators were analyzed (Figure 11), and the results indicated that significant positive correlations existed between the brown rice rate, milled rice rate, and head milled rice rate. Furthermore, significant positive correlations between the chalky kernel rate and chalkiness of rice were also observed. Positive correlations between the gel consistency of rice and protein and significant negative correlations with straight-chain starch were also recorded. In contrast, a significant negative correlation was found between the amylose content and protein content of rice. This suggests that the nutritional quality of rice can be increased by reducing the formation of chalkiness and increasing the gel consistency.

### 3.5. Ecological, Environmental, and Economic Benefits of the RDC

We calculated the input and output values of the RDC in terms of the cost of facility protection, labor, electricity, feed, crayfish, and ducklings over a year to assess the economic benefits of this technique. It was found that the rice yield was higher in the RDC than in the SSR, by approximately 7.9% (Table 3). Consequently, the profit of the RDC was 136.61% and 18.35% higher than that of the SSR and DSR, respectively. In comparison to the DSR, although its smaller rice planting area for crayfish farming resulted in a certain degree of reduction in the total rice production, the ripe rice in the RDC can obtain a higher market price. Together with the extra income from the ducks and crayfish, the total net income of the RDC was much higher than those of the other treatments [69,70].

>Ecosystem services (ESs) are commonly used to assess the comprehensive and quantitative values of ecosystems [71,72,73]. The total net ES value of the RDC in this work was calculated to be 1.02 × 10^8^ ¥ hm^−2^, while those of the SSR and DSR were 7.00 × 10^4^ and 9.75 × 10^4^ ¥ hm^−2^, respectively (Table 4). Conversion from the SSR and DSR to the RDC increased the total net ES values by 44.99% and 6.00%, respectively. It is worth noting that the RDC may have some negative impacts on arable land. The RDC generates some negative values, but the positive values are much greater.

## 4. Conclusions

The present study suggests that the RDC improves the properties of the soil in the selected area, including soil fertility, soil humus content, and soil enzyme activity. Relative to the SSR, the RDC reduces the loss of TN and TP from agricultural runoff and does not contribute HM pollution to paddy soils. In addition, the RDC can improve the quality of rice and increase the economic income of farmers. Several limitations also need to be considered, including the combination of rice and ducks in terms of quantity and potential organic pollutants derived from duck feed in the environment. To better promote this technique, the local government should offer more opportunities for training and guidance to farmers so that they can maximize the environmental friendliness and high productivity of the RDC and reduce the application of chemical fertilizers and pesticides. Overall, the RDC system can serve as a sustainable method for the management of agricultural nonpoint-source pollution, and produce high quality rice that might also provide substantial benefits to farmers and consumers. The RDC system is a promising option for cultivating rice in southern China.

## Figures and Tables

**Table 1:** Detailed descriptions of different treatments.

Groups	Processing	Fertilizer Application (kg hm^−2^)	Pesticide Application	Total Planting Area (hm^2^)
Rice–duck–crayfish ecological co-culture system (RDC)	First stage: paddy field (crayfish: 375 kg hm^−2^); later stage: paddy field (195 ducks hm^−2^).	Base fertilizer: 300; supplementary fertilizer: 75	-	5.34
Idle field (CK)	-	-	-	0.45
Single-season rice planting system (SSR)	Inactive in the first period; late-season rice planting.	Base fertilizer: 600; supplementary fertilizer: 150	3 kg hm^−2^	0.67
Double-season rice planting system (DSR)	Planting rice at the first stage; later planting of late rice	Base fertilizer: 600; supplementary fertilizer: 150 (one season)	3 kg hm^−2^	1.34

Notes: “-” indicates no use.

**Table 2:** Effects of different systems on the Nemerow index (*P_N_*).

Indicators	P_-Cu_	P_-Zn_	P_-Cd_	P_-Pb_	P_-As_	P_-Cr_	Nemerow Index (*P_N_*)
RDC	0.28	0.58	0.75	0.90	0.75	0.09	0.75
CK	0.25	0.60	0.63	0.85	0.72	0.11	0.71
SSR	0.29	0.81	0.83	0.98	0.69	0.09	0.82
DSR	0.23	0.62	0.76	1.03	0.67	0.14	0.83

**Table 3:** Effects of different systems on the annual yield and economic benefit of paddy fields.

Treatment	Rice			*Procambarus clarkii*. Duck		Total Output Value (¥ hm^−2^)	Total Input Value (¥ hm^−2^)	Profit (¥ hm^−2^)
	Yield (t hm^−2^)	Output Value (¥ hm^−2^)	Input Value (¥ hm^−2^)	Output Value (Million hm^−2^)	Input Value (¥ hm^−2^)			
RDC	8.39 ± 0.17	2.07 × 10^4^	1.30 × 10^4^	3.38 × 10^4^	1.67 × 10^4^	5.45 × 10^4^	3.00 × 10^4^	2.45 × 10^4^
SSR	7.96 ± 0.11	2.19 × 10^4^	1.16 × 10^4^	-	-	2.19 × 10^4^	1.16 × 10^4^	1.03 × 10^4^
DSR	8.16 ± 0.20	2.13 × 10^4^	1.21 × 10^4^	-	-	4.49 × 10^4^	2.42 × 10^4^	2.07 × 10^4^

**Table 4:** Ecosystem service value (¥ hm^−2^ year^−1^) of each system.

Ecosystem Service (¥ hm^−2^ Year^−1^)	RDC	SSR	DSR
Food supply	5.45 × 10^4^	2.19 × 10^4^	4.49 × 10^4^
Gas regulation	2.99 × 10^3^	2.83 × 10^3^	5.81 × 10^3^
Temperature regulation	3.30 × 10^4^	3.30 × 10^4^	3.30 × 10^4^
Flood-control storage	3.87 × 10^3^	3.78 × 10^3^	3.78 × 10^3^
Soil maintenance	1.00 × 10^4^	9.82 × 10^3^	1.13 × 10^4^
Waste disposal	4.87 × 10^2^	3.83 × 10^2^	3.83 × 10^2^
Destruction of arable land	−1.83 × 10^3^	0.00	0.00
GHG emissions	−1.50 × 10^3^	−1.70 × 10^3^	−3.4 × 10^3^
Total value	1.02 × 10^5^	7.00 × 10^4^	9.58 × 10^4^

## Data Availability

Not applicable.

## Supplementary Materials

The following supporting information can be downloaded at: https://www.mdpi.com/article/10.3390/ijerph20032006/s1. Supporting information includes further details on chemical analysis and calculation method; Figure S1 and Tables S1–S7 as noted in the text.
