# Supplementary material for: Initial Studies on the Effect of the Rice–Duck–Crayfish Ecological Co-Culture System on Physical, Chemical, and Microbiological Properties of Soils: A Field Case Study in Chaohu Lake Basin, Southeast China
Source: Int J Environ Res Public Health. 2023 Jan 21;20(3):2006. doi: 10.3390/ijerph20032006 (PMC9916220; doi:10.3390/ijerph20032006)
Supplement: Supplementary file 1 [file ijerph-20-02006-s001.zip › ijerph-2139640-supplementary.pdf]

Supplementary Information

Chemical analysis

Soil chemical properties determination method

The samples were air-dried, and the soil chemical properties (pH, soil organic matter (SOM), total nitrogen (TN), total phosphorus (TP), alkali-hydrolysable nitrogen (AN), available phosphorus (AP), and available potassium (AK)) were measured according to reported methods [1]. Specifically, soil pH was determined using a soil: water ratio of 1: 5. The soil organic matter content (SOM) was determined using the potassium-dichromate ($K_2Cr_2O_7$) digestion method. TN was analyzed by using Kjeldahl digestion method. TP was measured by using molybdate-ascorbic acid method after oxidative digestion of soil samples with H_2SO_4-$HClO_4$. AN was detected by the alkali solution diffuse method, AP in soil was tested by the $NaHCO_3$ (pH 8.5) extraction-phosphomolybdate blue spectrophotometric method, and AK in soil was obtained by the NH_4OAc extraction-flame photometer method.

Water quality indicators determination method The TP and DTP levels in the water samples were determined by the ammonium molybdate spectrophotometric method [2]. Herein, potassium persulfate was used as the oxidizing agent; for TP, the unfiltered water samples were directly digested, while for DTP, the water samples filtered with 0.45 μm filter membrane were digested; and then ammonium molybdate was added into the above digestions; finally quantified by UV-vis spectrophotometer (UV-360, Shimadzu, Japan).

The TN concentration was determined by the alkaline potassium persulfate digestion method [3]. The TN was determined by high-temperature digestion of water samples using alkaline potassium persulfate and measured by UV spectrophotometry (UV-360, Shimadzu, Japan).

Enzyme activity determination method

Enzyme activity was measured as described by Guan et al. [4]. Specifically, soil urease was determined using the indophenol blue colorimetry method, and the enzyme activity was expressed in μg of NH_3-N produced in 1 g soil sample after 24 h. Disodium phenyl phosphate colorimetry was used for acid phosphatase, and activity was expressed as mg phenol kg^{-1} soil. The catalase activity was determined by the potassium permanganate titration method, and its activity was expressed as the volume (mL) of 0.02 mol L^{-1} potassium permanganate consumed

by 1 g of soil after 20 minutes.

HM content determination and BCR sequential extraction

As part of digestion, the pretreated soil sample (0.2000 ± 0.0005 g) accurately weighed was separately digested by a mixture of acids (subboiling grade) consisting of 5 mL of HF, 5 mL of HNO_3, and 3 mL of $HClO_4$ in Teflon crucibles. Before measuring the elements, the samples were diluted to 80 g in a PET bottle with an internal standard (1 g of indium) and ultra-pure water. The concentrations of five HMs were analyzed via an inductively coupled plasma-mass spectrometer (Agilent 7500 Series, Agilent, USA).

To extract the four fractions of five HMs, a three-step sequential extraction procedure was applied based on the modified BCR [5]: the acid-soluble/exchangeable fraction (F1), reducible fraction (F2), oxidizable fraction (F3), and residual fraction (F4). The detailed extraction procedures are listed in Table S1. Following the extraction process, the supernatant filtrate was obtained through a 0.45-μm fiber membrane before determining the available HMs using ICP–MS.

Table S1 The extraction procedure, reagents and extraction components of European Community Bureau of Reference (BCR) standard method

Step	Target phase	Extraction agent and procedures
1	Exchangeable/acid-soluble fraction-F1	Duplicate samples, 1.0 g+40 mL of 0.11 M CH_3COOH, Shaking for 16 h then centrifuge
2	Reducible fraction-F2	Step 1 residue sample, 40 mL NH_2OH-HCl 0.5 M (pH=1.5), Shaking for 16h then centrifuge
3	Oxidizable fraction-F3	Step 2 residue sample, 10 mL H_2O_2 (8.8 M) heat to 85°C for 1 h (Repeated once) and then add 50 mL CH_3COONH_4 1 M (pH=2), Shaking for 16 h then centrifuge
4	Residual fraction-F4	Step 3 residue sample (0.2 g), digested by a mixture of acids including 5 mL HNO_3, 5 mL HF, and 3 mL $HClO_4$ in Teflon beakers, heating on a hot plate equipment

Calculation method

runoff nitrogen and phosphorus loss

The runoff nitrogen and phosphorus losses are equal to the sum of the product of the nitrogen and phosphorus concentrations in each runoff water and the volume of runoff water for the entire monitoring cycle (a complete growing season). The calculation formula is as follows.

$$Q=\sum_{i=1}^{n}(C_i\times V_i) \tag{1}$$

where Q is the amount of nitrogen and phosphorus lost in g hm^{-2}.

C_i is the concentration of nitrogen and phosphorus in runoff water in mg L^{-1}.

V_i is the volume of runoff water in m^3 hm^{-2}.

Heavy metal pollution index method

In this study, we used the heavy metal contamination index method to assess the risk of soil heavy metals in paddy fields in RDC evaluation. The specific calculation is shown below.

$$P_i=\frac{C_n^i}{S_n^i} \tag{2}$$

$$P_N=\sqrt{\frac{P_{imax}^2+P_{iave}^2}{2}} \tag{3}$$

where$P_i$is the single contamination index of heavy metal *i* in the soil.

C_n^i-Measured levels of heavy metal *i* in soil, mg kg^{-1}.

S_n^i-The evaluation standard content of heavy metal *i*, mg kg^{-1}.

P_N-Pollution composite pollution index.

P_{imax}-The maximum value of the individual pollution index of all pollutants.

P_{iave}-The average of the individual pollution indices of all pollutants. Based on P_i or P_N, soil contamination levels are classified into five grades, as shown in Table S2.

Table S2 Soil environmental grade-nemero composite pollution index

P_N**Scope**	**Pollution level**	**Evaluation results**
(0, 0.7]	I	Safe, non-polluting
(0.7, 1.0]	II	Alert line, not yet contaminated
(1.0, 2.0]	III	Light pollution
(2.0, 3.0]	IV	Moderate pollution

(3.0, +∞)	V	Severe pollution

Ecosystem service value evaluation method

The latest Common International Classification for Ecosystem Services (CICES) version 5.1 was used for ES classification and evaluation [6]. The ESs were therefore divided into three categories: provisioning, regulation and maintenance, and cultural activities. Table S3 summarizes the ESs associated with rice-based agroecosystems. ES functions include food supply, gas regulation, temperature regulation, flood control storage, waste disposal, GHG emissions, soil maintenance, destruction of arable land, and development of tourism. Due to the lack of data availability, other functions, such as tourism development and nonpoint source pollution, were excluded from this study.

TableS3 Methods related to rice-based agroecosystems that provide biotic ecosystem services

Terminology used in this study	Value evaluation method
**Positive value**	
Food supply	Market value method
Gas regulation	Afforestation cost method; Carbon tax method
Temperature regulation	Replacement cost method
Flood-control storage	Shadow project method
Soil maintenance	Opportunity cost method; Market value method
Waste disposal	Replacement cost method
**Negative value**	
Destruction of arable land	Market value method
GHG emissions	Afforestation cost method; Carbon tax method

Table S4 Effects of different paddy field cultivation patterns on soil physical properties of paddy field

Depth/cm	Treatment	Physical properties	
		Bulk density/ g cm^{-3}	Total porosity/%
0-10	RDC	1.21±0.05ab	54.26±1.73c
	CK	1.19±0.03b	58.31±1.12a
	SSR	1.27±0.08a	55.78±4.71b
	DSR	1.29±0.11a	49.44±4.14d
10-20	RDC	1.40±0.15ab	47.09±3.73c

CK	1.35±0.03b	52.99±1.07a
SSR	1.34±0.05b	50.64±3.32b
DSR	1.45±0.12a	46.55±1.05c

Note: Different letters indicate that the difference between modes reached 0.05 significant level, the same below.

Table S5 Effects of different patterns on soil HA-$\Delta \log k$ and HA-E4/E6 in paddy soil

Treatment	*Δlog k*		*E4/E6*	
	**0-10cm**	**10-20cm**	**0-10cm**	**10-20cm**
RDC	0.698 ± 0.02 a	0.712 ± 0.02 a	4.272 ± 0.11 a	4.115 ± 0.01 b
CK	0.671 ± 0.01 b	0.681 ± 0.02 b	4.148 ± 0.12 b	4.107 ± 0.08 c
SSR	0.649 ± 0.01 d	0.637 ± 0.02 d	3.978 ± 0.01 d	3.982 ± 0.05 d
DSR	0.661 ± 0.05 c	0.651 ± 0.05 c	4.085 ± 0.10 c	4.128 ± 0.05 a

Table S6 Nitrogen and phosphorus loss

**Treatment**	**TN loss (kg hm^{-2})**	**NH_4^+-N loss (kg hm^{-2})**	**NO_3^--N loss (kg hm^{-2})**	**TP loss (kg hm^{-2})**	**DTP loss (kg hm^{-2})**
RDC	12.61±0.81b	7.71±0.36b	2.59±0.20a	0.98±0.15b	0.66±0.05a
SSR	16.67±0.65a	11.91±0.45a	2.08±0.15b	1.25±0.11a	0.78±0.08a

Table S7a Effects of different cultivation modes on rice milling quality

**Treatment**	**Brown rice rate (%)**	**Milled rice rate (%)**	**Head milled rice rate (%)**
RDC	78.32±0.59a	72.61±0.61a	68.42±0.26a
SSR	75.46±0.64b	70.53±0.58b	64.87±0.39b

Table S7b Effects of different cultivation modes on rice appearance quality

Treatment	Chalky rice rate (%)	Chalkiness degree (%)	Grain length (mm)	Grain width (mm)	Length–width ratio
RDC	9.85±0.32b	1.84±0.14b	4.75±0.12a	2.86±0.08a	1.66±0.09a
SSR	11.92±0.41a	2.23±0.08a	4.82±0.09a	2.85±0.11a	1.69±0.06a

Table S7c Effects of different cultivation modes on rice cooking and nutrition quality

Treatment	Protein (%)	Amylose content (%)	Gel consistency (mm)	Alkali spreading value
RDC	8.23±0.26a	15.62±0.25b	77.37±1.16a	5.17±0.24a
SSR	7.61±0.19b	17.08±0.31a	72.63±1.62b	5.22±0.18a

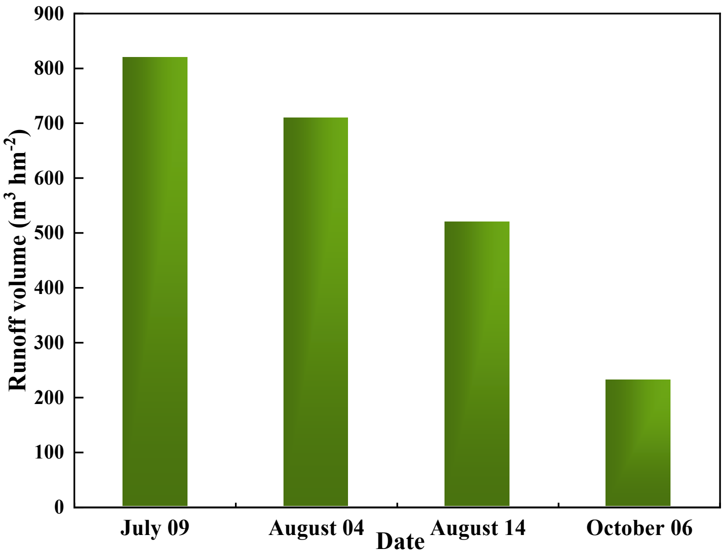

Figure S1. Runoff amount and runoff-yielding time.

References

1. Bao, S.D. *Soil and Agricultural Chemistry Analysis*; China Agricultural Press: Beijing, China, 2000.
2. *GB 11893-89*; Water quality—Determination of total phosphorus—Ammonium molybdate spectrophotometric method; Ministry of Ecology and Environment of China: Beijing, China, 1989.
3. *GB 11894-89*; Water quality—Determination of total nitrogen—Alkaline potassium persulfate digestion-UV spectrophotometric method; Ministry of Ecology and Environment of China: Beijing, China, 1989.
4. Guan, S.Y.; Zhang, D.; Zhang, Z. Soil enzyme and its research methods; Agricultural Press: Beijing, China, 1986.
5. Pueyo, M.; Mateu, J.; Rigol, A.; Vidal, M.; López-Sánchez, J.F.; Rauret, G. Use of the modified BCR three-step sequential extraction procedure for the study of trace element dynamics in contaminated soils. *Environ. Pollut.* **2008**, *152*, 330–341. https://doi.org/10.1016/j.envpol.2007.06.020.
6. Xu, Q.; Liu, T.; Guo, H.; Dou, Z.; Gao, H.; Zhang, H. Conversion from rice–wheat rotation to rice–crayfish coculture increases net ecosystem service values in Hung-tse Lake area, east China. *J. Clean. Prod.* **2021**, 319, 128883. https://doi.org/10.1016/j.jclepro.2021.128883.
